# Low-Dose Paclitaxel Inhibits Tumor Cell Growth by Regulating Glutaminolysis in Colorectal Carcinoma Cells

**DOI:** 10.3389/fphar.2017.00244

**Published:** 2017-05-04

**Authors:** Chaoxiang Lv, Hao Qu, Wanyun Zhu, Kaixiang Xu, Anyong Xu, Baoyu Jia, Yubo Qing, Honghui Li, Hong-Jiang Wei, Hong-Ye Zhao

**Affiliations:** ^1^State Key Laboratory for Conservation and Utilization of Bio-Resources in Yunnan, Yunnan Agricultural UniversityKunming, China; ^2^Key Laboratory of Agricultural Biodiversity and Plant Disease Management of China Education Ministry, Yunnan Agricultural UniversityKunming, China; ^3^College of Plant Protection, Yunnan Agricultural UniversityKunming, China; ^4^College of Pharmacy and Chemistry, Dali UniversityDali, China

**Keywords:** low-dose PTX, cell growth, lactate production, glutaminolysis, colorectal carcinoma cells

## Abstract

Paclitaxel (PTX) is a natural alkaloid isolated from the bark of a tree, *Taxus brevifolia*, and is currently used to treat a variety of tumors. Recently, it has been found that low-dose PTX is a promising treatment for some cancers, presenting few side effects. However, antitumor mechanisms of low-dose PTX (<1 nM) have rarely been illuminated. Here we report a new antitumor mechanism of low-dose PTX in colorectal carcinoma cells. We treated colorectal carcinoma HCT116 cells with PTX at 0.1 and 0.3 nM for 0, 1, 2, or 3 days, and found that low-dose PTX inhibits cell growth without altering cell morphology and cell cycle. There was a significant decrease of pH in culture media with 0.3 nM PTX for 3 days. Also, lactate production was significantly increased in a dose- and time-dependent manner. Furthermore, expression of glutaminolysis-related genes *GLS, SLC7A11* and *SLC1A5* were significantly decreased in the colorectal carcinoma cells treated with low-dose PTX. Meanwhile, protein expression levels of p53 and p21 increased significantly in colorectal carcinoma cells so treated. In summary, low-dose PTX down-regulated glutaminolysis-related genes and increased their lactate production, resulting in decreased pH of tumor microenvironments and inhibition of tumor cell growth. Up-regulation of p53 and p21 in colorectal carcinoma cells treated with low-dose PTX also contributed to inhibition of tumor cell growth.

## Introduction

Paclitaxel (PTX) has been used for treating various malignancies. It stabilizes polymerized microtubules and enhances microtubule assembly and thus, arrests the cell cycle and induces apoptosis (Barbuti and Chen, 2015). Despite PTX being often indicated as a first-line therapy for treating some cancers, some side effects of high-dose PTX including peripheral neuropathy and anaphylactic hypersensitivity reactions have limited its clinical application (Banerji et al., 2014; Duggett et al., 2016). Recently, low-dose metronomic PTX chemotherapy has attracted increasing attention as an alternative scheme for treatment of some cancers (Lien et al., 2013) because of its high efficiency, low toxicity and reduced drug resistance in clinical settings (Loven et al., 2013; Gong et al., 2014). Anticancer mechanisms of low-dose metronomic PTX include inhibition of progress and metastasis of cancer through its strong anti-angiogenic and anti-lymphangiogenic activities, rather than by inducing tumor cell apoptosis (Jiang et al., 2010; Bocci et al., 2013). In addition, our previous studies revealed that 1 and 3 nM PTX inhibit the growth of colorectal carcinoma cells by down-regulating c-Myc, and those concentrations did not damage the intestinal crypt epithelial IEC-6 cells (Li et al., unpublished). However, antitumor mechanisms of low-dose PTX (<1 nM) have seldom been reported.

Tumor cell proliferation requires sufficient energy to synthesis macromolecules, and need to be allocated into appropriate metabolic pathways to produce energy and maintain cell growth (Gentric et al., 2016). It has been reported that glucose metabolism and glutamine metabolism together account for most of the carbon and nitrogen metabolism in mammalian cells (Polet et al., 2016). Glycolysis is a major energy metabolism pathway for tumor cells, and targeting this pathway is a potential strategy to inhibit cancer cell growth. The pathway involves multiple genes, for example, the solute carrier family 2 member 1 (*GLUT1*) gene brings extracellular glucose into the cytoplasm, hexokinase1 (*HK1*) genes phosphorylate intracellular glucose to form glucose-6-phosphate. Other genes, such as phosphoglycerate dehydrogenase (*PHGDH*) and pyruvate dehydrogenase kinase1 (*PDK1*), also play roles in glycolysis (Chen et al., 2016). Meanwhile, glutaminolysis also provides carbon and nitrogen to support biosynthetic homeostasis that cancer cells may exploit for growth (Altman et al., 2016). In this process, glutaminase (*GLS*), which converts glutamine to glutamate, plays a key role in cancer cell metabolism, growth, and proliferation (Kung et al., 2011). The glutamate dehydrogenase1 (*GDH*) encodes a trifunctional protein that is associated with the enzymatic activities of glutaminolysis (Koo and Yoon, 2015). The solute carrier family 1 member 5 (*SLC1A5*) is related to glutamine uptake (Bolzoni et al., 2016). The solute carrier family 7 member 11 (*SLC7A11*) is responsible for glutamate release in glutaminolysis (Kalivas, 2009). Therefore, a comprehensive understanding of the regulation of metabolic fluxes in tumor cell proliferation would contribute to the development of new strategies to treat cancer.

Colorectal carcinoma, a very common malignant tumor of the digestive system, represents a major cause of global cancer-related deaths (Abdifard et al., 2016). Despite several treatment options for colorectal carcinoma, response rates are relatively low and recurrence is high (Zhou et al., 2016). Chemotherapy, which has limited efficacy and poor prognoses, is currently the primary option for treating colorectal cancer (Jagadish et al., 2016). It has been reported that PTX is effective in the treatment of colorectal cancer (Zhang et al., 2016). Recent studies have shown that PTX can affect glucose metabolism in triple negative breast cancer (Stewart et al., 2016), and that PTX redirects metabolic reprogramming from glycolysis to oxidative phosphorylation, leading to effective suppression of ovarian cancer stem cells (Shen et al., 2015).

However, few studies have investigated effects of low-dose PTX (<1 nM) on glycolysis and glutaminolysis in colorectal carcinoma cells. Here we found that low-dose PTX could inhibit cell growth by regulating glutaminolysis as well as p53 and p21 expression in colorectal carcinoma cells. These findings also illustrate some antitumor mechanisms of low-dose PTX action on cancer cells.

## Materials and Methods

### Reagents and Antibodies

Paclitaxel was from Sigma–Aldrich Inc. (St. Louis, MO, USA). Antibodies against p21 and p53 were from Abcam (Burlingame, CA, USA). HK1, PHGDH, GLS, SLC7A11, SLC1A5, and GDH were from Absin Bioscience Inc. (Shanghai, China). β-actin was from Sigma–Aldrich Inc. (St. Louis, MO, USA).

### Cell Lines and Cell Cultures

Human colorectal carcinoma cell line HCT116 was from the Cell Collection of the Chinese Academy of Sciences (Shanghai, China) and cultured in DMEM-F-12 medium (HyClone, Logan, UT, USA) supplemented with heat-inactivated 10% fetal bovine serum (Gibco, Carlsbad, CA, USA) and 100 IU/mL penicillin (Solarbio, Beijing, China). The cells were seeded into a gelatin-coated 75-cm^2^ flask and cultured in 10 mL of medium at 37°C in a humidified atmosphere of 5% CO_2_ in air.

### Cell Morphological Observation

Exponentially growing HCT116 cells were transferred to 12-well plates and cultured at 37°C in a 5% CO_2_ atmosphere. Cells were treated with 0.1 and 0.3 nM PTX for 1–3 days. When the cells were at 60 to 70% confluence, they were rinsed twice with PBS, and the supernatant was discarded. Then, images were taken using an OLYMPUS IX 71 microscope (10 × 10) (OLYMPUS, Tokyo, Japan).

### Colony Formation Assay

Cells in the logarithmic growth phase were digested into a single-cell suspension with a trypsin-EDTA (Solarbio, Tongzhou District, Beijing, China) solution, and then 10 mL of the cell suspension was seeded onto 9 cm culture plates (NEST, Wuxi, Jiangsu, China) at a density of 20 cells/mL. After adherence, these cells were treated with PTX (0.1 and 0.3 nM) for 72 h and then cultured for 15 days. Thereafter, the cells were fixed in formaldehyde and stained with 10% Giemsa stain (Solarbio, Tongzhou District, Beijing, China). After multiple washes, the plates were air dried and imaged. Individual clones were counted, and statistical analyses were performed.

### Cell Survival by the MTT Assay

The 3- (4, 5 - dimethylthiazol -2 - yl) - 2, 5 - diphenyltetrazolium bromide (MTT) colorimetric assay was used to determine cytotoxicity of PTX. HCT116 cells were plated at a density of 1000 and 2000 cells/well in 96-well plates and were incubated either with or without PTX at 0.1 or 0.3 nM for 3 days. Then, 50 μL of MTT 1 mg/mL (Sigma–Aldrich) tetrazolium substrate was added to each well, and plates were incubated for an additional 4 h at 37°C. The resulting violet formazan precipitate was solubilized by adding 100 μL of 50% N,N-dimethylformamide. All of these plates were shaken for 5 min and read immediately at 578 nm using a model 550 Micro Plate Reader (Bio-Rad, Richmond, CA, USA).

### Flow Cytometric Analysis of the Cell Cycle

The cells were plated into 6-well plates and incubated for 1 and 3 days with nutrient solution containing various concentrations of PTX (0, 0.1, and 0.3 nM). Briefly, the cells were collected and fixed in 0°C 70% ethanol and stored at -20°C. The cells were then washed and resuspended in cold PBS and incubated at 37°C for 30 min with 10 mg/mL RNase and 1 mg/mL propidium iodide (Sigma–Aldrich, USA). DNA content analysis was performed by flow cytometry (BD, San Diego, CA, USA). The percentage of cells in different cell cycle phases was determined with Cell Quest acquisition software (BD Biosciences Pharmingen, USA).

### Lactate Assay and pH Determination

Lactate generation was analyzed, following the manufacturer’s instructions. In brief, HCT116 cells were treated with low-dose PTX (0.1 and 0.3 nM) for 0, 1, 2, or 3 days. Then, the culture media supernatant was collected for the detection of lactate generation using a Lactate Assay Kit purchased from Sigma–Aldrich, Inc. (MAK064, St. Louis, MO, USA) and an EnVision Multilabel Reader. Lactate generated was calculated as follows: Lactate generation (ng/μL) = (amount of lactic acid in unknown sample from standard curve/sample volume added into the wells) ^∗^ lactate molecular weight.

Culture media samples treated with PTX were collected at the indicated time points as described above to directly measure their pH with a meter from Sartorius (PB-10, Göttingen, Germany).

### RNA Isolation and Quantitative Real-Time PCR

HCT116 cells were treated with 0.1 and 0.3 nM PTX for 0, 1, 2, or 3 days. Total RNA was isolated using TRIpure reagent (BioTek, China), according to the manufacturer’s instructions. The cDNA was synthesized from total RNA using a PrimeScript RT reagent Kit with g Eraser (TaKaRa, Japan). The obtained cDNA was used as a template in SYBR green-based quantitative-polymerase chain reaction (q-PCR) (CFX-96, Bio-Rad, USA). The mRNA expression levels of glycolysis-related genes and glutaminolysis-related genes were assessed by q-PCR. β-actin was used for normalization. Primers are listed in **Table 1**.

**Table 1:** Primer sequences for q-PCR.

Gene Name	Primer Sequence (5′to 3′)
LDHA	F: 5′-ATTTCACTGTCTAGGCTACAACA-3′
	R: 5′-TTAATACCATCCAGCATCAGG-3′
HK1	F: 5′-GGAGCCACCACTCACCCTACT-3′
	R: 5′-GGAGCCCATTGTCCGTTACTT-3′
PHGDH	F: 5′-ACCCTGCAATGCTGCCTACCA-3′
	R: 5′-ACATGCTGCTTCCACGCTTCC-3′
PDK1	F: 5′-ATCACCAGGACAGCCAATACA-3′
	R: 5′-TCCTCGGTCACTCATCTTCAC-3′
GLUT1	F: 5′-GACATTCAAGGCATTTCTATCACAT-3′
	R: 5′-CGACTTCAGGCACATAACCTCTTT-3′
GDH	F: 5′-AATGCTGGAGGAGTGACAGTATCTTA-3′
	R: 5′-CTTGGAACTCTGCCGTGGGTA-3′
GLS	F: 5′-GAGTACTGAGCCCTGAAGCAGTTCG-3′
	R: 5′GGAGACCAGCACATCATACCCA-3′
SLC7A11	F: 5′-AGAGGGTCACCTTCCAGAAAT-3′
	R: 5′-AGATAAATCAGCCCAGCAACT-3′
SLC1A5	F: 5′-GAGAAATATCTTCCCTTCCAACCTGG-3′
	R: 5′-CCAAAGACGATGGCAAACACTACC-3′
β-actin	F: 5′-CTTAGTTGCGTTACACCCTTTCTTG-3′
	R: 5′-TCACCTTCACCGTTCCAGTTTT-3′

### Protein Extraction and Immunoblotting

After HCT116 cells were treated with PTX at the indicated times and concentrations, cells were washed twice with PBS and collected. Then, total protein concentrations of cell lysates were determined with a BCA Protein Assay kit (Beyotime, Shanghai, China). Protein samples (total protein loading of 100 μg) were separated by 10% SDS–PAGE and transferred onto PVDF membranes. These membranes were incubated for 30 min in 5% BSA buffer (Solarbio, Beijing, China) with gentle shaking to block non-specific binding before incubation with the diluted primary antibody (p53: 1:200, p21: 1:200, β-actin: 1:5000) overnight at 4°C. Subsequently, membranes were incubated with 5000-fold diluted secondary antibody (Santa Cruz, CA, USA) for 90 min at room temperature. The membrane was washed three times in PBS, for 10 min each time. Then, the membrane was treated for 3 min in the dark with reagent from an Easysee Western Blot Kit (Transgene, Alsace, France).

### Statistical Analysis

Statistical comparisons were performed using Student’s *t*-test. Quantitative data were expressed as the means ± SD. *p* < 0.05 was considered significant. ^∗^*p* < 0.05 and ^∗∗^*p* < 0.01 versus controls.

## Results

### Effect of Low-Dose PTX on the Morphology and Viability of HCT116 Cells

**FIGURE 1:**
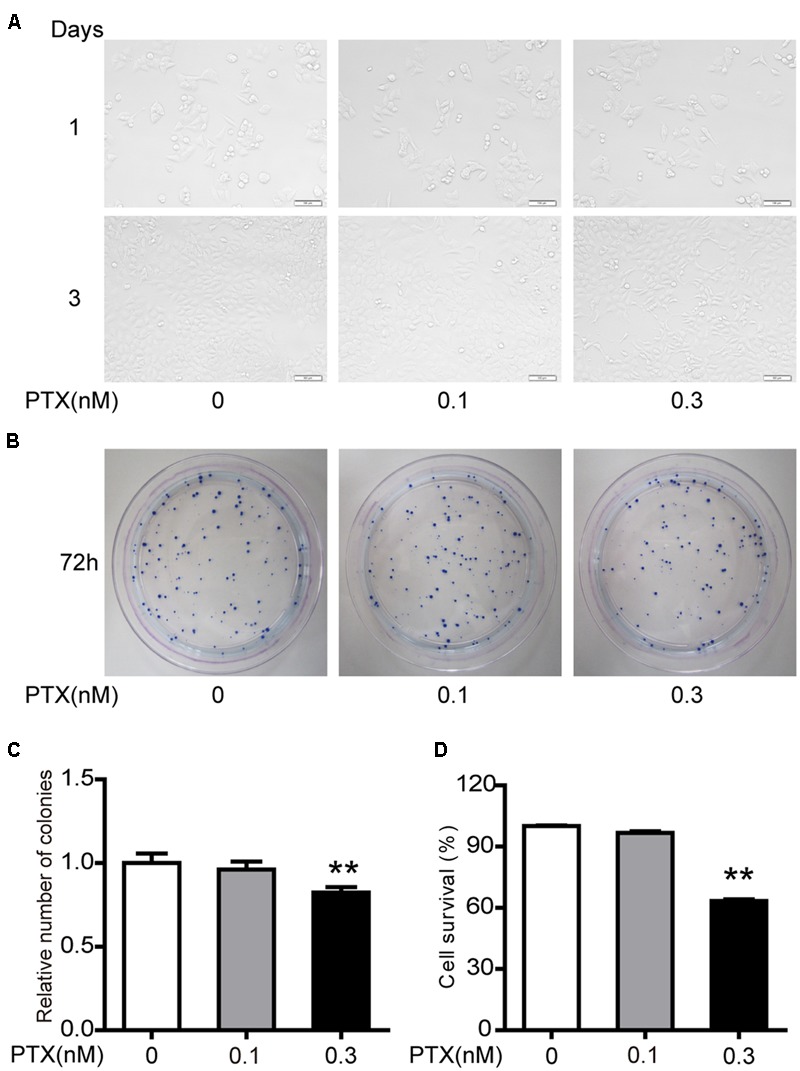
**Effect of low-dose PTX on the sensitivity of HCT116 cells. (A)** Photomicrographs of HCT116 cells exposed to 0.1 and 0.3 nM PTX for 1 or 3 days (original magnification, 100×). **(B)** Cell viability was detected by colony formation assay. **(C)** Results of cellular colony formation rate was expressed as a fold change. **(D)** Quantification of cell viability by MTT assay. Results are shown as mean ± SD. Date are representative of three independent experiments. (^∗∗^*p* < 0.01).

Our previous studies demonstrated that 1 and 3 nM PTX had an impact on morphology and viability of colorectal carcinoma HCT116 cells (Li et al., unpublished). Here, we further investigated whether lower doses of PTX had similar effects. Here we found that the low-dose PTX did not alter morphology of HCT116 cells (**Figure 1A**), however, it significantly decreased colony-forming ability of these cells (**Figures 1B,C**). Furthermore, the MTT assay further indicated that low-dosage PTX significantly inhibited viability of HCT116 cells in a dose-independent manner (**Figure 1D**). These findings indicate that low-dose PTX exerts a significant influence on the proliferation of HCT116 cells.

### Effect of Low-Dose PTX on the Cell Cycle in HCT116 Cells

**FIGURE 2:**
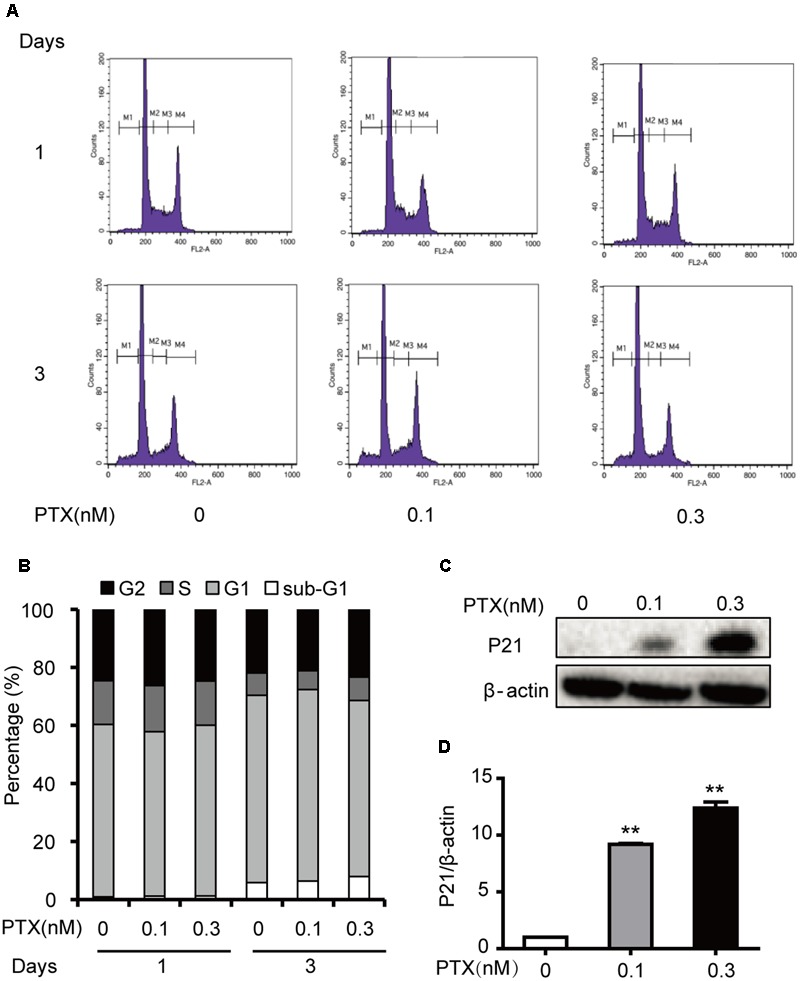
**Effects of low-dose PTX on the HCT116 cell cycle. (A)** HCT116 cells were exposed to PTX at 0.1 and 0.3 nM for 1 or 3 days and then analyzed with flow cytometric FCM. (M1: sub-G_1_; M2: G_0_/G_1_; M3: S; M4: G_2_/M). **(B)** The histogram of the cell cycle distribution of HCT116 cells treated with PTX at the indicated concentrations and time. **(C)** The total protein expression level of p21 in HCT116 cells. HCT116 cells were treated with PTX at the indicated concentrations for 3 days. Whole cell extracts were prepared, and equal amounts of protein were separated on SDS–PAGE and examined by Western blot. The control for protein loading with β-actin is shown. **(D)** Quantification of p21 total protein expression level. Data represent the means ± SD, *n* = 3 independent experiments. ^∗^*p* < 0.05 and ^∗∗^*p* < 0.01 versus control.

Our previous results indicated that 1 and 3 nM PTX blocked the cell cycle at the G_0_/G_1_ phase, which inhibited colorectal carcinoma cell proliferation (Li et al., unpublished). To investigate effects of low-dose PTX on the cell cycle in HCT116 cells, we also performed an analysis of the cell cycle using flow cytometry (**Figure 2A**). We found that the sub-G_1_, G_1_, S and G_2_ phases of HCT-116 cells were not significantly changed by treatment at indicated concentrations and times (**Figure 2B**).

Interestingly, these PTX treatments increased levels of p21 protein by 5.87- and 7.34-fold, in a dose-dependent manner (**Figures 2C,D**). As an inhibitor of cyclin D/cdk complexes, p21 can influence the cell cycle (Orlando et al., 2015), but our results indicated that the cycle of treated cells did not change. Based on the above findings, we surmised that low-dose PTX acts through another mechanism, other than blocking the cell cycle, to inhibit colorectal carcinoma cell growth.

### Effect of Low-Dose PTX on Lactate Production and the mRNA Expression of LDHA

**FIGURE 3:**
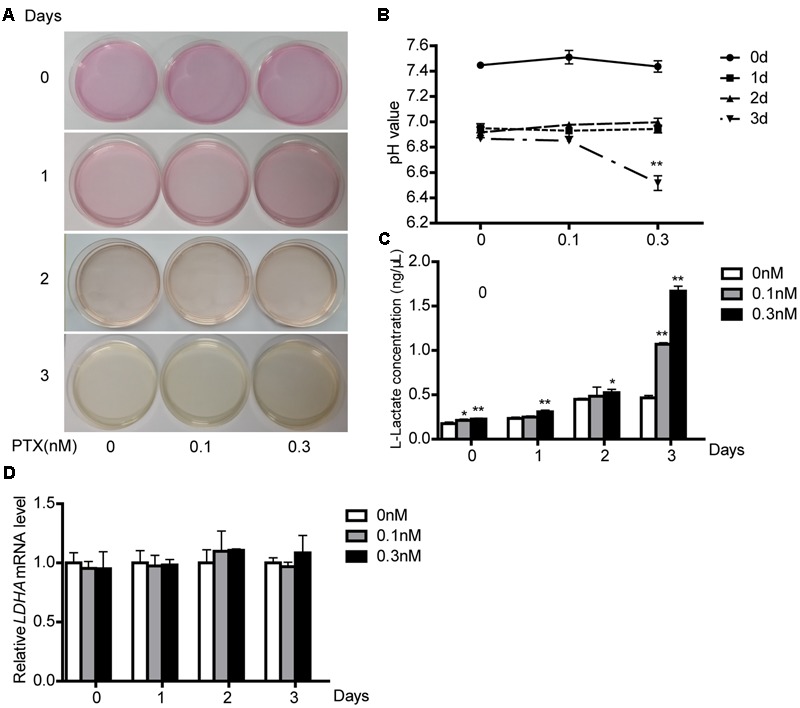
**Effect of low-dose PTX on lactate production and LDH activity. (A)** HCT116 cells were seeded in 10 mm^2^ dishes and treated with 0.1 and 0.3 nM PTX for 0, 1, 2, or 3 days. HCT116 cells cultured in medium only served as the control. **(B)** The medium pH. HCT116 cells treated with low-dose PTX at the indicated concentrations and time. **(C)** Media were collected and subjected to a lactate assay. **(D)** After measuring lactate, the cells were harvested, and the mRNA expression level of *LDHA* was determined by q-PCR. Data represent the means ± SD, *n* = 3 independent experiments. ^∗^*p* < 0.05 and ^∗∗^*p* < 0.01 versus control.

When HCT116 cells were treated as described **Figure 3A**, we found that the culture media gradually turned yellow through time. This media color indicated higher acidity. We thus collected the culture media as described above and determined its pH value. Culture media pH decreased through time (**Figure 3B**).

Tumor cell proliferation is susceptible to alterations in microenvironment pH (Bolzoni et al., 2016). To explore causes of reduced pH, we measured its lactate concentration, because lactate is a major source of acidity in cell metabolism (Seheult et al., 2017). We found that low-dose PTX increased lactate production with dose and time (**Figure 3C**), consistent with the lowered pH of the medium. In glucose metabolism, lactate production is primarily affected by the lactate dehydrogenase enzyme (LDH). However, the mRNA expression level of *LDHA* was not different in cells treated with or without PTX (**Figure 3D**), which suggests that PTX induces lactate production through a mechanism other than increased expression of *LDHA*.

### Effect of Low-Dose PTX on Glycolysis-Related Genes in HCT116 Cells

**FIGURE 4:**
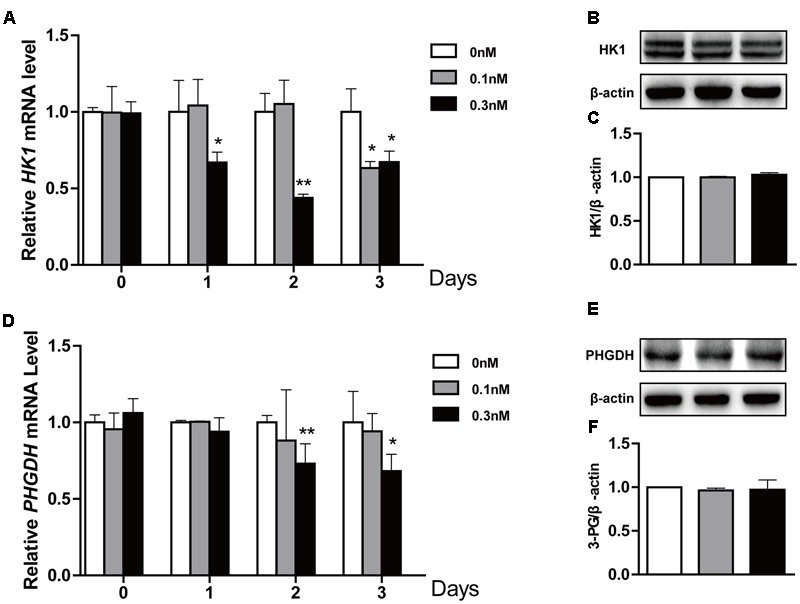
**Effect of low-dose PTX on the expression level of glycolysis-related genes in HCT116 cells.** HCT116 cells were exposed to PTX at 0.1 and 0.3 nM for 0, 1, 2, or 3 days, followed by gene expression levels detected by quantitative real-time PCR assay and Western blot. **(A,D)** The mRNA expression of *HK1* and *PHGDH* genes. **(B,E)** The protein expression of HK1 and PHGDH in cells treated with 0.1 and 0.3 nM PTX for 3 days. **(C,F)** Quantification of the protein expression of HK1 and PHGDH. Data represent the means ± SD, *n* = 3 independent experiments. ^∗^*p* < 0.05 and ^∗∗^*p* < 0.01 versus control.

Lactate is final product of glycolysis, and this pathway is regulated by *HK1, PHGDH, GLUT1* and *PDK1* among others (Soga, 2013; Wu et al., 2014). Thus, to determine whether lactate production was related to glycolysis, we measured expression levels of glycolysis-related genes in treated cells using qPCR and Western blot. We found that the low-dose PTX significantly decreased mRNA expression of glycolysis-related genes *HK1* and *PHGDH* in time-dependent manners (**Figures 4A,D**), however, the protein expression of these genes did not change (**Figures 4B,C,E,F**). Moreover, the mRNA expression of glycolysis-related genes *PDK1* and *GLUT1* did not change (**Supplementary Figure S1**). Therefore, pathways other than glycolysis appear to be involved in the PTX-induced lactate production.

### Effect of Low-Dose PTX on Glutaminolysis-Related Genes in HCT116 Cells

**FIGURE 5:**
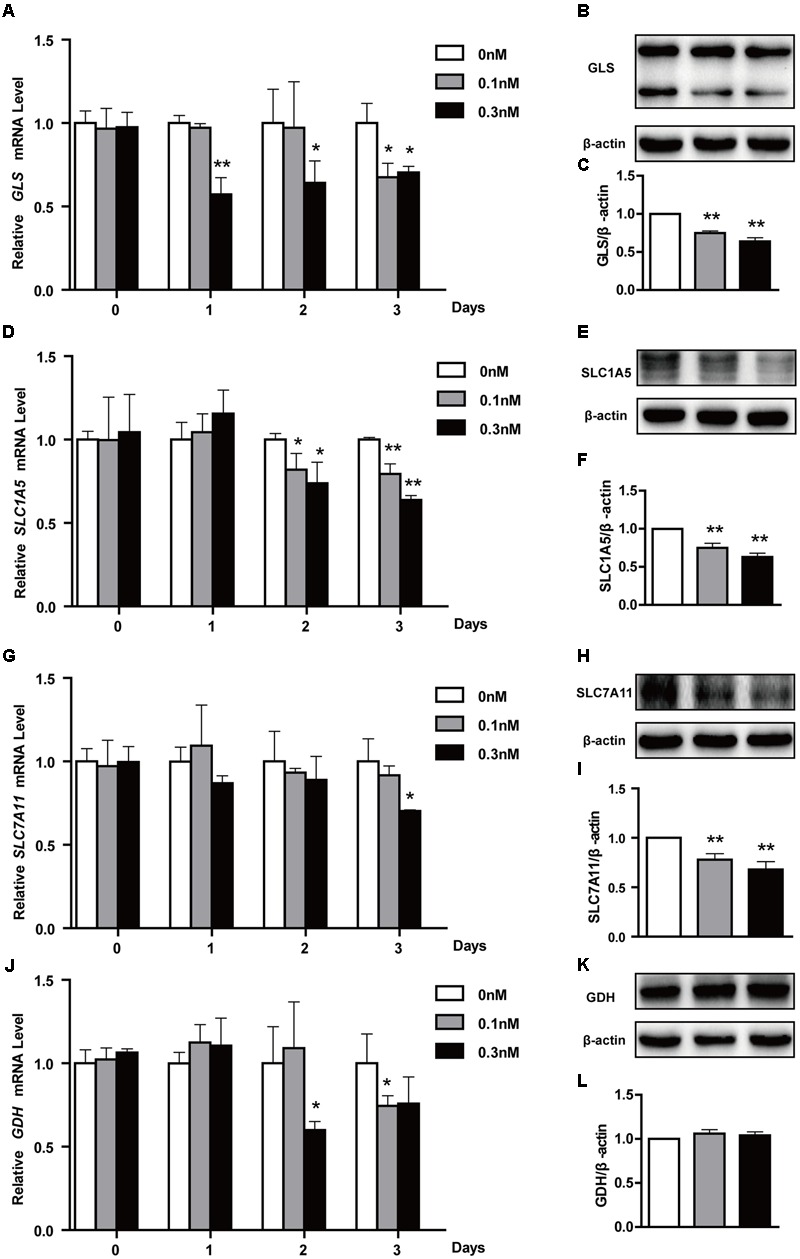
**Effects of low-dose PTX on mRNA expression of glutaminolysis-related genes in HCT116 cells.** HCT116 cells were treated with 0.1 and 0.3 nM PTX for 0, 1, 2, or 3 days, and then mRNA and protein expression of glutaminolysis-related genes was detected by quantitative real-time PCR assay and Western blot. **(A,D,G,J)** The mRNA expression of *GLS, SLC1A5, SLC7A11* and *GLUD1* genes. **(B,E,H,K)** The protein expression of GLS, SLC1A5, SLC7A11 and GLUD1 in cells treated with 0.1 and 0.3 nM PTX for 3 days. **(C,F,I,L)** Quantification of the protein expression, GLS, SLC1A5, SLC7A11 and GLUD1. Data represent the means ± SD, *n* = 3 independent experiments. ^∗^*p* < 0.05 and ^∗∗^*p* < 0.01 versus control.

To maintain a robust increase in nutrient uptake, in addition to increasing glycolysis, tumor cells commonly activate glutaminolysis. *GLS, GDH, SLC7A11* and *SLC1A5* genes play an important role in glutamate transport and metabolism (Locasale, 2013). To further explore whether glutaminolysis was related to lactate production, we also treated HCT116 cells as described above and found that low-dose PTX significantly decreased the mRNA of glutaminolysis-related genes *GLS* and *SLC1A5* in a time-dependent manner, *SLC7A11* for 3 days at 0.3 nM PTX (**Figures 5A,D,G**). And the protein expression level of *GLS, SLC1A5* and *SLC7A11* genes significantly decreased in dose-dependent manner (**Figures 5B,C,E,F,H,I**). In addition, the mRNA expression of *GDH* significantly decreased in a time-dependent manner, whereas the protein expression had no change (**Figures 5J–L**). Our results showed that the low-dose PTX significantly decreased the expression level of glutaminolysis-related genes, promoting lactate production.

### Effect of Low-Dose PTX on the Expression of p53 in HCT116 Cells

**FIGURE 6:**
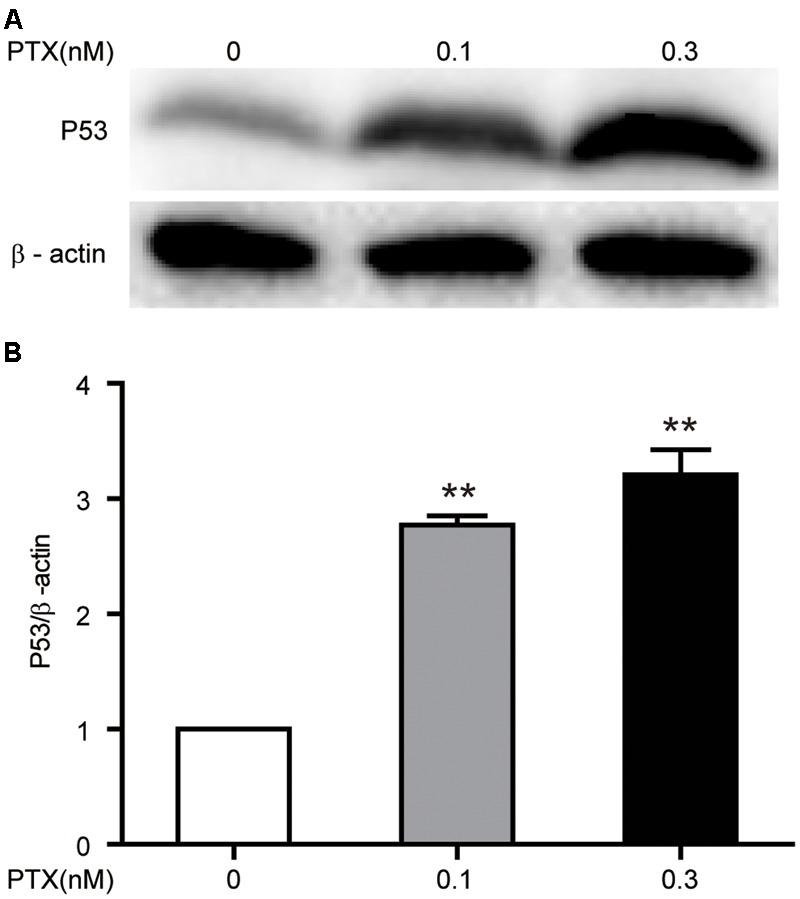
**Effect of low-dose PTX on the expression of p53 in HCT116 cells.** HCT116 cells were treated with low concentrations of PTX, as described above, and then expression levels of p53 was detected by Western blot. **(A)** The total protein expression of the p53 gene. **(B)** Quantification of the p53 total protein expression. Data represent the means ± SD, *n* = 3 independent experiments. ^∗^*p* < 0.05 and ^∗∗^*p* < 0.01 versus control.

Published reports show that PTX inhibits tumor cell growth through up-regulation of tumor-suppressive p53 (Choi and Yoo, 2012). In addition, based on the above finding of an increase in the protein expression level of p21, which is a downstream gene of p53, we further verified that low-dose PTX had an impact on cell growth through regulation of p53 in PTX-treated HCT116 cells. We detected the protein expression level of p53 in treated cells by Western blot and found that low-dose PTX significantly increased expression level of p53, by 2.34- to 2.88-fold, in a dose-dependent manner (**Figures 6A,B**).

## Discussion

In general, PTX could inhibit proliferation of tumor cell through blocking cell cycle G0/G1 or G2/M phases (Othman et al., 2001). However, we found that low-dose PTX (0.3 nM) inhibited the proliferation of HCT116 cells without cell-cycle change by up-regulating expression of p21 proteins (**Figures 1, 2A**). This may be due to p21 inhibiting cellular growth by pathways not regulating cell cycle, for instance, during notch 1 activation, p21 suppressed E2F1-dependent Wnt4 expression to inhibit tumor cell proliferation (Ren et al., 2014). p21 also binds to STAT3 inhibiting STAT3-dependent gene expression and preventing cell growth (Siveen et al., 2014). In addition, we also found that the expression of p53 as the upstream gene of p21 was significantly up-regulated by PTX dosage (**Figures 6A,B**). Therefore, we suggest that low-dose PTX inhibits tumor cell proliferation by significantly increasing p21 protein expression by up-regulation of p53. Detailed mechanisms will require further study.

Interestingly, we found that low-dose PTX was able to significantly increase lactate production and decrease media pH (**Figures 3A,B**). It has been reported that increased lactate secretion can reduce pH and thus affect enzymes and coenzymes function, with negative effects on cell metabolism providing energy and materials for tumor cells (Evelyn et al., 2006). Moreover, the lower pH would affect membrane charge distributions, altering absorption of nutrients and the discharge of metabolites (Sanhueza et al., 2016). The pH value is an important parameter that significantly influences cell growth, recombinant protein production, cell metabolism and protein glycosylation (Trummer et al., 2006). Our results demonstrate that low-dose PTX affect the growth of HCT116 cells through lowered pH value.

Recently, it has been reported that many anticancer drugs could inhibit tumor cell growth through inhibition of glutaminolysis (Gill et al., 2016). Moreover, inhibition of glutaminolysis increased lactate production in several types of tumors (Draoui and Feron, 2011; Todaka et al., 2017). Consistent with these results, we found that low-dose PTX down-regulated expression of glutaminolysis-related genes (*GLS, SLC7A11* and *SLC1A5*) and increased lactate production in HCT116 cells (**Figures 3C, 5**). These findings suggest that the low-dose PTX could inhibit HCT116 cells by targeting glutaminolysis, however, mechanisms remain to be elucidated.

**FIGURE 7:**
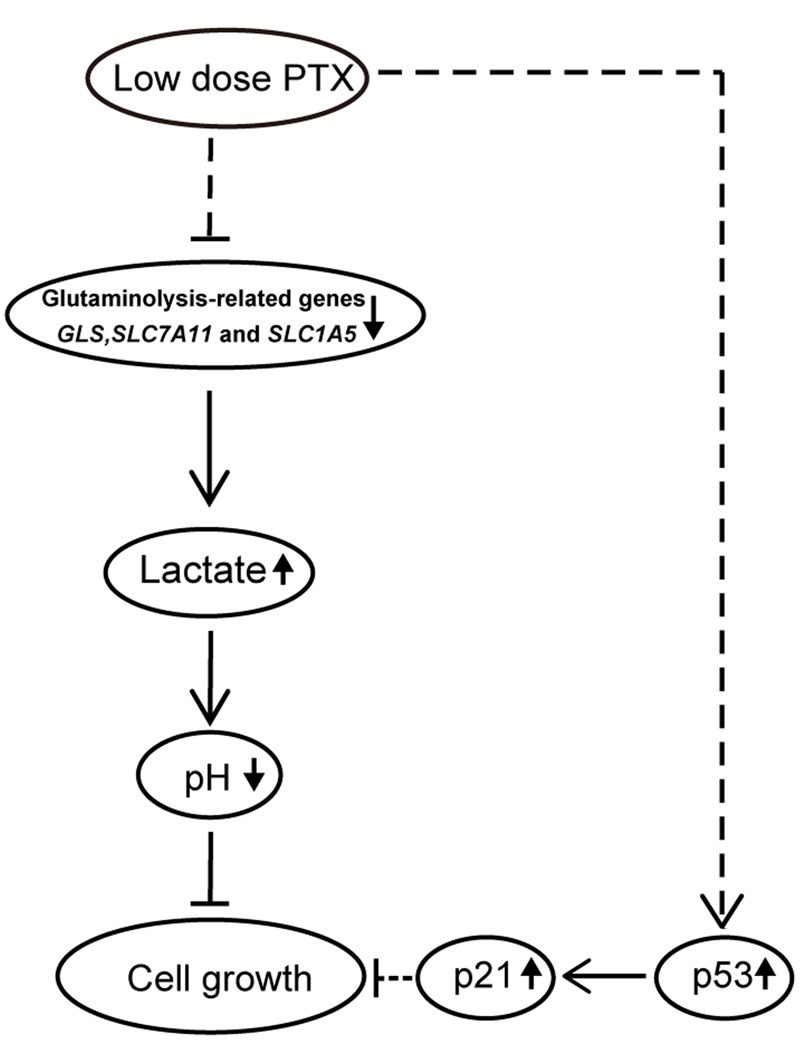
**Schematic pathway for low-dose PTX inhibition of cell growth in colorectal carcinoma cells.** In this model, down-regulation of glycolysis-and glutaminolysis-related genes by low-dose PTX through an unknown pathway halted cell growth. The blockage of cell growth might involve the p53 signaling pathways.

**Figure 7** is a schematic representation of proposed molecular basis for the low-dose PTX inhibition of tumor cell growth by regulating glutaminolysis in HCT116 cells. We found that low-dose PTX down-regulated glutaminolysis-related genes (*GLS, SLC7A11* and *SLC1A5*) and increased lactate production, resulting in decreased pH of the tumor microenvironment which inhibited tumor cell growth. Additionally, up-regulation of p53 and p21 in colorectal carcinoma cells also contributed to this inhibition. We thus present a theoretical basis for further study of anticancer mechanisms of low-dose PTX. In addition, it will facilitate the discovery of “smart” therapeutics that can inhibit tumor energetics and viability.

## Author Contributions

H-YZ and H-JW conceived and designed the experiments. LC, HQ, WZ, AX, BJ, YQ, and HL performed the experiments. H-YZ, H-JW, and KX analyzed the data. H-YZ and WZ wrote the paper. All authors reviewed the manuscript.

## Conflict of Interest Statement

The authors declare that the research was conducted in the absence of any commercial or financial relationships that could be construed as a potential conflict of interest.

## Supplementary Material

FIGURE S1 **Effect of low-dose PTX on the expression lK1 and GLUT1 in HCT116 cells.** HCT116 cells were exposed to PTX at 0.1 and 0.3 nM for 0, 1, 2, or 3 days, and then gene expression levels were detected by a quantitative real-time PCR assay. **(A)** The mRNA expression of HK1 gene. **(B)** The mRNA expression of GLUT1 gene. Data represent the means ± SD, *n* = 3 independent experiments. ^∗^*p* < 0.05 and ^∗∗^*p* < 0.01 versus control.
