# Supplementary figures and images for: Low-Dose Paclitaxel Inhibits Tumor Cell Growth by Regulating Glutaminolysis in Colorectal Carcinoma Cells
Source: Front Pharmacol. 2017 May 4;8:244. doi: 10.3389/fphar.2017.00244 (PMC5415623; doi:10.3389/fphar.2017.00244)

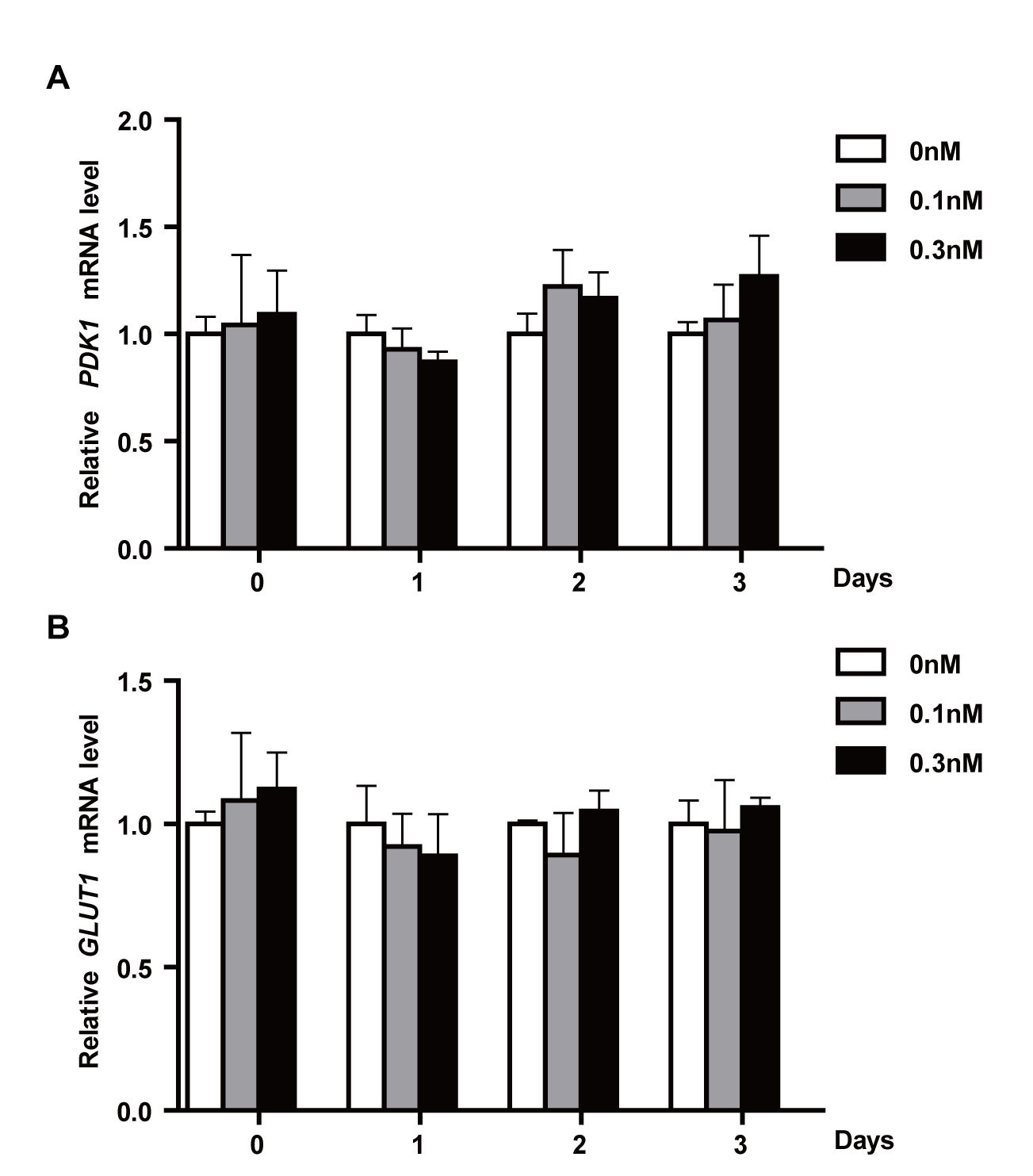

Supplement: FIGURE S1 — Effect of low-dose PTX on the expression lK1 and GLUT1 in HCT116 cells. HCT116 cells were exposed to PTX at 0.1 and 0.3 nM for 0, 1, 2, or 3 days, and then gene expression levels were detected by a quantitative real-time PCR assay. (A) The mRNA expression of HK1 gene. (B) The mRNA expression of GLUT1 gene. Data represent the means ± SD, n = 3 independent experiments. ∗p < 0.05 and ∗∗p < 0.01 versus control. [file Image_1.TIF]
